# Comparison of laminoplasty and posterior fusion surgery for cervical ossification of posterior longitudinal ligament

**DOI:** 10.1038/s41598-021-04727-1

**Published:** 2022-01-14

**Authors:** Hiroaki Nakashima, Shiro Imagama, Toshitaka Yoshii, Satoru Egawa, Kenichiro Sakai, Kazuo Kusano, Yukihiro Nakagawa, Takashi Hirai, Kanichiro Wada, Keiichi Katsumi, Kengo Fujii, Atsushi Kimura, Takeo Furuya, Tsukasa Kanchiku, Yukitaka Nagamoto, Yasushi Oshima, Narihito Nagoshi, Kei Ando, Masahiko Takahata, Kanji Mori, Hideaki Nakajima, Kazuma Murata, Shunji Matsunaga, Takashi Kaito, Kei Yamada, Sho Kobayashi, Satoshi Kato, Tetsuro Ohba, Satoshi Inami, Shunsuke Fujibayashi, Hiroyuki Katoh, Haruo Kanno, Yuanying Li, Hiroshi Yatsuya, Masao Koda, Yoshiharu Kawaguchi, Katsushi Takeshita, Morio Matsumoto, Masashi Yamazaki, Atsushi Okawa, Japanese Multicenter Research Organization for Ossification of the Spinal Ligament, Hiroaki Nakashima, Shiro Imagama, Toshitaka Yoshii, Satoru Egawa, Kenichiro Sakai, Kazuo Kusano, Yukihiro Nakagawa, Takashi Hirai, Kanichiro Wada, Keiichi Katsumi, Kengo Fujii, Atsushi Kimura, Takeo Furuya, Tsukasa Kanchiku, Yukitaka Nagamoto, Yasushi Oshima, Narihito Nagoshi, Kei Ando, Masahiko Takahata, Kanji Mori, Hideaki Nakajima, Kazuma Murata, Shunji Matsunaga, Takashi Kaito, Kei Yamada, Sho Kobayashi, Satoshi Kato, Tetsuro Ohba, Satoshi Inamia, Shunsuke Fujibayashi, Hiroyuki Katoh, Haruo Kanno, Masao Koda, Yoshiharu Kawaguchi, Katsushi Takeshita, Morio Matsumoto, Masashi Yamazaki, Atsushi Okawa

**Affiliations:** 1 Department of Orthopaedic Surgery, Nagoya University Graduate School of Medicine, 65 Tsurumaicho, Showa Ward, Nagoya, Aichi 466-8550 Japan; 2 Department of Orthopaedic Surgery, Tokyo Medical and Dental University, 1-5-45 Yushima, Bunkyo Ward, Tokyo, 113-8519 Japan; 3 Department of Orthopaedic Surgery, Saiseikai Kawaguchi General Hospital, 5-11-5 Nishikawaguchi, Kawaguchishi, Saitama 332-8558 Japan; 4 Department of Orthopaedic Surgery, Kudanzaka Hospital, 1-6-12 Kudanminami, Chiyodaku, 102-0074 Japan; 5 Department of Orthopaedic Surgery, Wakayama Medical University Kihoku Hospital, 219 Myoji, Katsuragi-cho, Itogun, Wakayama 649-7113 Japan; 6 Department of Orthopaedic Surgery, Hirosaki University Graduate School of Medicine, 5 Zaifucho, Hirosaki, Aomori, 036-8562 Japan; 7 Department of Orthopaedic Surgery, Niigata University Medicine and Dental General Hospital, 1-754 Asahimachidori, Chuo Ward, Niigata, Niigata 951-8520 Japan; 8 Department of Orthopaedic Surgery, Faculty of Medicine, University of Tsukuba, 1-1-1 Tennodai, Tsukuba, Ibaraki 305-8575 Japan; 9 Department of Orthoaedics, Jichi Medical University, 3311-1 Yakushiji, Shimotsuke, Tochigi 329-0498 Japan; 10 Department of Orthopaedic Surgery, Chiba University Graduate School of Medicine, 1-8-1 Inohana, Chuo Ward, Chiba, Chiba 260-8670 Japan; 11 Department of Orthopaedic Surgery, Yamaguchi University School of Medicine, 111 Minami Kogushi, Ube, Yamaguchi 755-8505 Japan; 12 Department of Orthopaedic Surgery, Osaka Rosai Hospital, 1179-3 Nagasonecho, Sakaishi, Osaka 591-8025 Japan; 13 Department of Orthopaedic Surgery, Faculty of Medicine, The University of Tokyo, 7-3-1 Hongo, Bunkyo-ku, Tokyo, 113-0033 Japan; 14 Department of Orthopaedic Surgery, School of Medicine, Keio University, 35 Shinanomachi, Shinjuku Ward, Tokyo, 160-8582 Japan; 15 Department of Orthopaedic Surgery, Faculty of Medicine and Graduate School of Medicine, Hokkaido University, Kita 15, Nishi 7, Sapporo, 060-8638 Japan; 16 Department of Orthopaedic Surgery, Shiga University of Medical Science, Tsukinowa-cho, Seta, Otsu, Shiga 520-2192 Japan; 17 Department of Orthopaedics and Rehabilitation Medicine, Faculty of Medical Sciences, University of Fukui, 23-3 Matsuoka Shimoaizuki, Eiheiji-cho, Yoshida-gun, Fukui, 910-1193 Japan; 18 Department of Orthopaedic Surgery, Tokyo Medical University, 6-7-1 Nishishinjuku, Shinjuku-ku, Tokyo, 160-0023 Japan; 19 Department of Orthopaedic Surgery, Imakiire General Hospital, 4-16 Shimotatsuocho, Kagoshimashi, 892-8502 Japan; 20 Department of Orthopaedic Surgery, Graduate School of Medicine, Osaka University, 2-2 Yamadaoka, Suita-shi, Osaka, 565-0871 Japan; 21 Department of Orthopaedic Surgery, Kurume University School of Medicine, 67 Asahi-machi, Kurume-shi, Fukuoka, 830-0011 Japan; 22 Department of Orthopaedic Surgery, Hamamatsu University School of Medicine, 1-20-1 Handayama, Hamamatsu, Shizuoka 431-3125 Japan; 23 Department of Orthopaedic Surgery, Graduate School of Medical Sciences, Kanazawa University, 13-1 Takara-machi, Kanazawa, 920-8641 Japan; 24 Department of Orthopaedic Surgery, University of Yamanashi, 1110 Shimokato, Chuo Ward, Yamanashi, 409-3898 Japan; 25 Department of Orthopaedic Surgery, Dokkyo Medical University School of Medicine, 880 Kitakobayashi, Mibu-machi, Shimotsuga-gun, Tochigi, 321-0293 Japan; 26 Department of Orthopaedic Surgery, Graduate School of Medicine, Kyoto University, 54 Kawahara-cho, Shogoin, Sakyo-ku, Kyoto, 606-8507 Japan; 27 Department of Orthopaedic Surgery, Surgical Science, Tokai University School of Medicine, 143 Shimokasuya, Isehara, Kanagawa 259-1193 Japan; 28 Department of Orthopaedic Surgery, Tohoku University School of Medicine, 1-1 Seiryomachi, Aoba Ward, Sendai, Miyagi 980-8574 Japan; 29 Department of Public Health, Fujita Health University School of Medicine, Aichi, Japan; 30 Department of Public Health and Health Systems, Nagoya University Graduate School of Medicine, Aichi, Japan; 31 Department of Orthopaedic Surgery, Faculty of Medicine, University of Toyama, 2630 Sugitani, Toyama, Toyama 930-0194 Japan

**Keywords:** Neurological disorders, Outcomes research

## Abstract

This prospective multicenter study, established by the Japanese Ministry of Health, Labour and Welfare and involving 27 institutions, aimed to compare postoperative outcomes between laminoplasty (LM) and posterior fusion (PF) for cervical ossification of the posterior longitudinal ligament (OPLL), in order to address the controversy surrounding the role of instrumented fusion in cases of posterior surgical decompression for OPLL. 478 patients were considered for participation in the study; from among them, 189 (137 and 52 patients with LM and PF, respectively) were included and evaluated using the Japanese Orthopaedic Association (JOA) scores, the JOA Cervical Myelopathy Evaluation Questionnaire (JOACMEQ), and radiographical measurements. Basic demographic and radiographical data were reviewed, and the propensity to choose a surgical procedure was calculated. Preoperatively, there were no significant differences among the participants in terms of patient backgrounds, radiographical measurements (K-line or cervical alignment on X-ray, OPLL occupation ratio on computed tomography, increased signal intensity change on magnetic resonance imaging), or clinical status (JOA score and JOACMEQ) after adjustments. The overall risk of perioperative complications was found to be lower with LM (odds ratio [OR] 0.40, p = 0.006), and the rate of C5 palsy occurrence was significantly lower with LM (OR 0.11, p = 0.0002) than with PF. The range of motion (20.91° ± 1.05° and 9.38° ± 1.24°, p < 0.0001) in patients who had PF was significantly smaller than in those who had LM. However, multivariable logistic regression analysis showed no significant difference among the participants in JOA score, JOA recovery rate, or JOACMEQ improvement at two years. In contrast, OPLL progression was greater in the LM group than in the PF group (OR 2.73, p = 0.0002). Both LM and PF for cervical myelopathy due to OPLL had resulted in comparable postoperative outcomes at 2 years after surgery.

## Introduction

Ossification of the posterior longitudinal ligament (OPLL), defined as heterotopic bone formation in the posterior longitudinal ligament^1^, is a common cause of degenerative cervical myelopathy (DCM)^2^. Surgical decompression is indicated in cases of moderate and severe myelopathy (modified Japanese Orthopaedic Association (JOA) score ≤ 14)^3^; however, patients with OPLL are at a higher risk of perioperative complications than patients with other forms of DCM^4^. Surgical intervention includes anterior, posterior, or combined approaches, but the posterior approach is predominantly chosen for surgical treatment with multilevel decompression (≥ 3 segments), because of the high rate of complications with the anterior approach and combined approaches^5^.

Posterior surgeries include laminoplasty (LP) and laminectomy with fusion (PF); however, the optimal technique remains debatable^6,7^. LP is recognized as a standard technique for the treatment of cervical multi-segment DCM, and a more satisfactory long-term outcome has been reported with LP in cases of cervical OPLL than with PF^8–10^. In contrast, however, some patients demonstrated poor results^11^, especially those who were K-line (−), and/or those with a high percentage of ossification occupation rate^12–14^. In addition, long-term follow-up has revealed increased ossification after LP, leading to reoperation^15,16^. PF, meanwhile, is another posterior procedure that has become widely used with the development of instruments^17^. PF has a low risk of kyphotic change and ossification progression after surgery, and physicians believe that PF is preferable to LP in cases with severe ossification and/or in those that are K-line (−)^6^, but sufficient scientific evidence has not been accumulated. Systematic review and meta-analysis comparing these procedures have shown equivalent postoperative results; however, the details are unclear because there is data only from a limited number of small-scale prospective studies^6^. In particular, the size and types of OPLL have a considerable impact on the severity of myelopathy and the postoperative course, which makes comparisons difficult^18,19^. Put simply, the two techniques must be compared under equal conditions, and with the fact that severe cases are more commonly dealt with via PF taken into account.

Thus, the objective of the present study was to compare postoperative outcomes between LP and PF for cervical OPLL in a propensity score-matched analysis adjusted for baseline factors and radiographical characteristics of spinal cord compression.

## Methods

**Figure 1:**
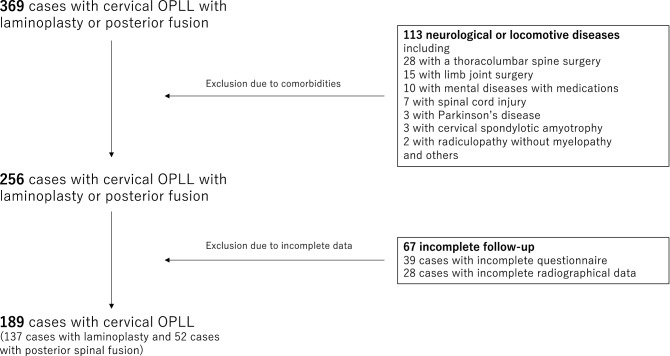
Patient selection flowchart.

This nationwide, multicenter, longitudinal study involved 28 academic institutions affiliated with the Japanese Multicenter Research Organization for Ossification of the Spinal Ligament formed by the Japanese Ministry of Health, Labour and Welfare. This study was approved by the Ethics Committee of Tokyo Medical and Dental University (M2000-1963), and conducted in accordance with the Declaration of Helsinki. All patients provided written informed consent when registered for possible participation. In total, 478 Japanese patients with cervical OPLL were prospectively enrolled between April 2015 and July 2017. Data were analyzed after obtaining approval from the Ethics Committee of all participating institutions. Patients were eligible for inclusion in the present study if they (1) were 20 years old or older; (2) had imaging evidence of OPLL on computed tomography (CT) and spinal cord compression on magnetic resonance imaging (MRI); and (3) had undergone surgery. All patients had surgical decompression for the cervical OPLL performed on them, and the attending surgeon determined the surgical approach and the number of levels to decompress. A total of 369 cases were initially enrolled in this study, among which 260 and 109 underwent LP and PF, respectively. We excluded 113 patients with preoperative comorbidities affecting neurological and lower**-**limb function, including 28 patients with a thoracolumbar spine surgery, 15 with limb joint surgery, 10 with mental illnesses requiring medications, 7 with spinal cord injury, 3 with Parkinson’s disease, 3 with cervical spondylotic amyotrophy, 2 with radiculopathy without myelopathy, and 45 other neurological or locomotive diseases. In addition, 39 patients with incomplete questionnaire data and 28 patients with incomplete radiographical followed-up data were excluded. Finally, the remaining 189 patients were included in the current study (Fig. 1 and Table 1).

**Table 1:** Demographic parameters, preoperative radiographical parameters and quality of life in the original sample (before weighting) of patients undergoing laminoplasty or posterior spinal fusion.

	Laminoplasty (n = 137)	Posterior spinal fusion (n = 52)	p
Age (years)	64.2 ± 11.6	63.9 ± 10.6	0.85
Sex (male ratio: %)	70.1	73.1	0.68
Body mass index	25.0 ± 3.7	26.4 ± 4.6	0.03
Smoking history (%)	32.8	44.2	0.15
Duration of symptoms (months)	46.5 ± 74.7	40.9 ± 55.7	0.63
**Levels of OPLL**			
C1–2 (%)	3.6	1.9	
C2–3 (%)	32.1	50.0	
C3–4 (%)	60.6	71.1	
C4–5 (%)	84.7	84.6	
C5–6 (%)	84.7	80.8	
C6–7 (%)	43.8	46.2	
C7–T1 (%)	5.1	26.9	0.009
Levels of surgery	3.4 ± 0.9	4.9 ± 1.5	< 0.0001
**Comorbidities**			
Diabetes mellitus (%)	29.2	40.4	0.14
Hypertension (%)	40.9	30.8	0.20
Malignancy (%)	5.8	5.8	0.99
Cerebrovascular disease (%)	7.3	3.8	0.39
Myocardial infarction (%)	2.9	7.7	0.16
Collagen disease (%)	0.7	3.8	0.17
**Drug**			
Anticoagulant	11.7	13.5	0.74
**Radiographical measurements**			
Cervical lordosis (°)	11.5 ± 11.4	6.5 ± 11.4	0.008
Range of motion (°)	26.3 ± 12.5	24.9 ± 14.7	0.50
K-line (+/−) (%)	73	59.6	0.08
Thickness of ossification (mm)	4.9 ± 1.6	6.3 ± 1.7	< 0.0001
Spinal canal occupation ratio > 60% (%)	6.6	34.6	< 0.0001
Increased signal intensity on MRI (%)	85.4	90.4	0.37
JOA score	11.1 ± 2.5	9.9 ± 3.2	0.007
**JOACMEQ**			
Cervical function	63.0 ± 27.7	53.8 ± 31.0	0.0501
Upper limb function	71.9 ± 21.9	64.1 ± 30.2	0.051
Lower limb function	56.3 ± 29.2	49.2 ± 30.6	0.14
Bladder function	73.3 ± 20.0	72.0 ± 26.7	0.71
Quality of life	44.6 ± 17.3	43.1 ± 20.0	0.62
**VISUAL analog scale**			
Pain or stiffness in the neck or shoulder	37.7 ± 32.0	50.0 ± 30.6	0.02
Tightness in the chest	9.7 ± 20.9	13.0 ± 20.3	0.03
Pain or numbness in the arms or hands	58.4 ± 31.1	68.8 ± 25.8	0.046
Pain or numbness from chest to toe	40.8 ± 33.4	51.4 ± 29.8	0.33

Summary statistics for continuous variables are means and standard deviations. *OPLL* ossification of posterior longitudinal ligament, *JOA* Japanese Orthopaedic Association, *JOACMEQ* Japanese Orthopaedic Association Cervical Myelopathy Evaluation Questionnaire, *MRI* magnetic resonance imaging.

### Surgical procedures

#### Laminoplasty

**Figure 2:**
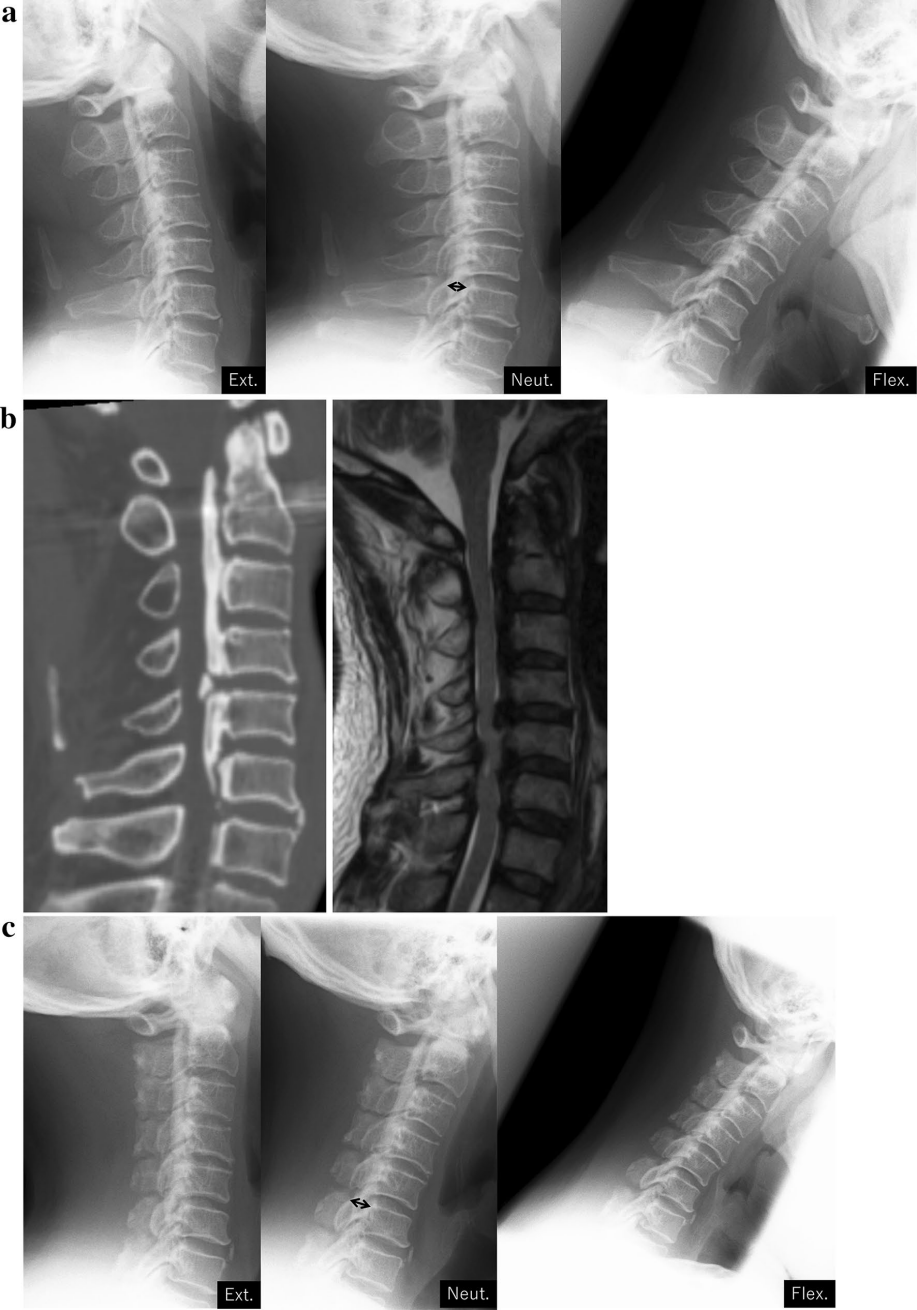
A representative case of cervical laminoplasty. (**a**): preoperative functional X-rays. The thickness of OPLL was 7.1 mm (double-arrow). The neutral position and range of motion at C2-C7 were 9° and 40°. (**b**) Preoperative CT and MRI sagittal images. (**c**) Two years postoperative functional X-rays. The thickness of OPLL was 9.4 mm (double-arrow). The neutral position and range of motion at C2–C7 were 3° and 25°.

Open- (25 cases) or French-door (112 cases) laminoplasty was performed^20,21^. A midline incision was made directly above the laminae, followed by detachment of the bilateral paravertebral muscles from the spinous processes. After cutting away the spinous processes, gutters were created on the bilateral laminae using a high-speed drill at the border of the laminae and facets. In the open-door laminoplasty cases, laminae on one side were completely cut to create an opening**,** and those on the other side were partially cut, preserving the ventral cortex, to prepare for a hinge. After this, the laminae were gradually opened. In the French-door laminoplasty cases, the center of the laminae was cut using a high-speed drill, in addition to bilateral gutters. After the halves of the laminae were elevated, a graft material was tied to bridge the bilateral edges of the laminae (Fig. 2).

#### Posterior spinal fusion

**Figure 3:**
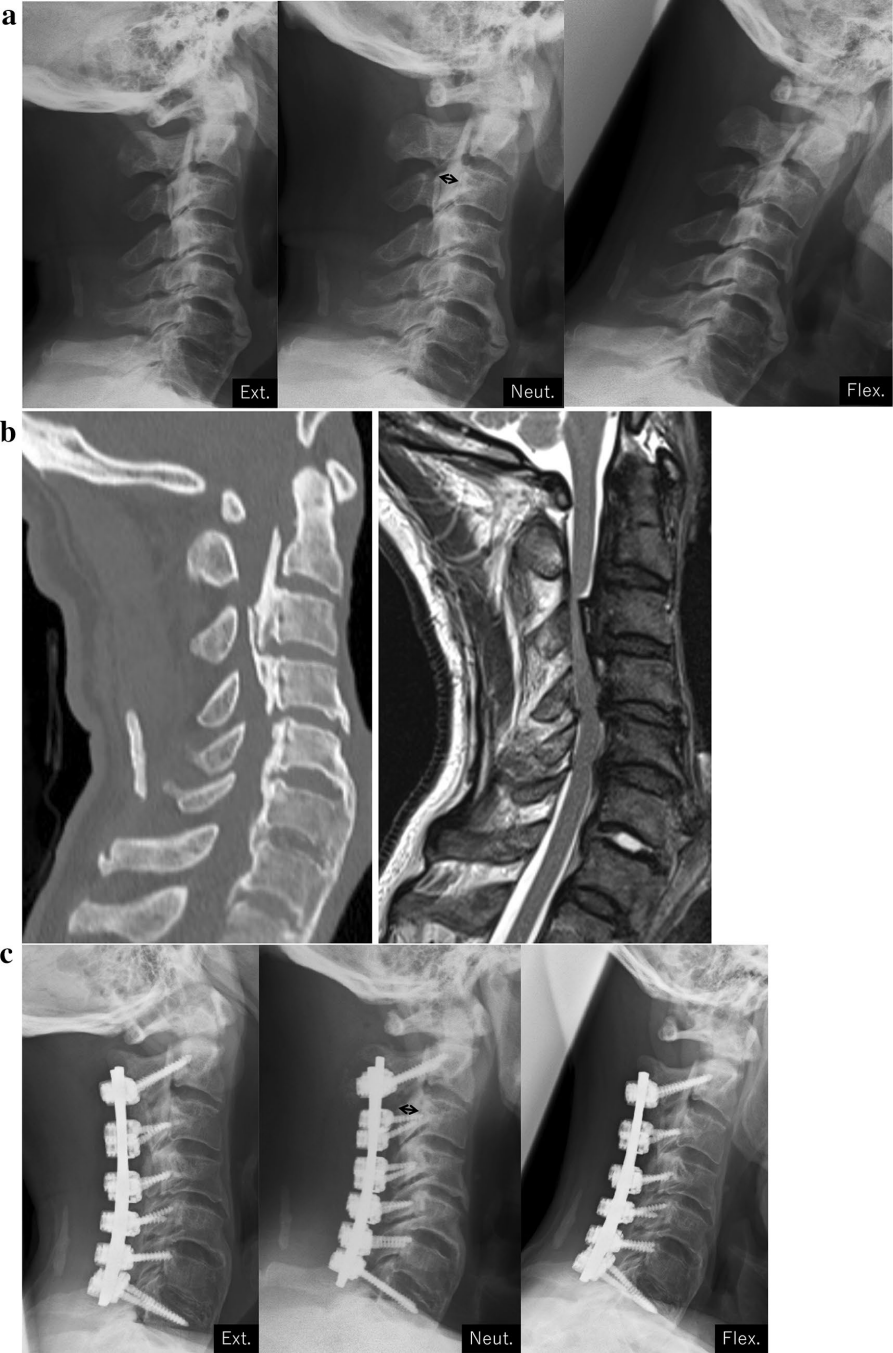
A representative case of cervical posterior fusion. (**a**) Preoperative functional X-rays. The thickness of OPLL was 8.0 mm (double-arrow). The neutral position and range of motion at C2–C7 were 11° and 20°. (**b**) Preoperative CT and MRI sagittal images. (**c**) 2 years postoperative functional X-rays. The thickness of OPLL was 7.4 mm (double-arrow). The neutral position and range of motion at C2–C7 were 19° and 0°.

Posterior spinal fusion using the pedicle screw and/or lateral mass screw system was performed^22^. With respect to surgical decompression, laminectomy or laminoplasty was performed in accordance with each facility’s policies (Fig. 3).

### Data collection

Data were collected for each participant, including demographic information, symptomatology, causative pathology, and surgical summary. Functional impairment, disability, and quality of life (QOL) were also evaluated preoperatively and at 24 months postoperatively. Basic demographic and clinical data, including age, sex, diabetes status, body mass index (BMI), smoking history, and disease duration, were collected for each patient.

### Evaluation

#### Clinical assessments

Clinical status was evaluated using the Japanese Orthopaedic Association (JOA) cervical score and the Japanese Orthopaedic Association Cervical Myelopathy Evaluation Questionnaire (JOACMEQ) preoperatively and at two years post-surgery. The recovery rate based on JOA scores was calculated as follows: (postoperative JOA − preoperative JOA)/(17 − preoperative JOA) × 100^23^. The minimum clinically important difference (MCID) for the JOA score and the JOA recovery rate was defined as 2.5 points and 52.8%, respectively^24^. The JOACMEQ, meanwhile, includes 24 questions covering the domains of cervical function, upper limb function, lower limb function, bladder function, and QOL^25^; scores for each domain were calculated according to official guidelines and range from 0 to 100 points, with a higher score indicating better health status. The JOACMEQ also incorporates visual analog scale (VAS) scores for pain or stiffness in the neck or shoulder, tightness in the chest, pain or numbness in the arms or hands, and pain or numbness from chest to toe. We evaluated improvement in each of the five JOACMEQ domains. Clinically significant improvement was confirmed if (1) the patient answered all the questions necessary to calculate the functional score for a domain and an increase of ≥ 20 points was obtained for that score, or (2) the functional score after treatment was > 90 points, even if answers for any unanswered questions were assumed to be the worst possible answers.

Each attending surgeon was also required to record all adverse events throughout the study period. A central panel of investigators classified each adverse event in relation to the surgery; any discrepancies among reviewers were resolved by consulting source documents. Perioperative complications were defined as surgery-related events occurring within 30 days of surgery.

#### Radiographical assessments

The most compressed level and the presence of a signal intensity change in the spinal cord were also investigated on mid-sagittal MRI.

The number of ossification levels (longitudinal extent of OPLL), K-line (positive or negative)^7^, JOA welfare classification (continuous, segmental, mixed, and circumscribed)^4^, spinal canal occupation ratio of OPLL on axial CT at the maximum cord compression level, and signal intensity change on T2 weight MRI were investigated.

The cervical lordotic angle (C2–7 angle) and range of motion (ROM) in flexion–extension were also measured**,** using tangential lines drawn on the posterior edge of the C2 and C7 vertebral bodies on lateral radiographs taken in a neutral position.

### Statistical analysis

Propensity score methods were used to estimate treatment effects from the observational data in the present study. A backward elimination logistic regression model was created to estimate the probability of treatment assignment, including all the relevant baseline variables with p-values less than 0.25. In order to balance the distribution of baseline variables between treatment groups, a pseudo sample was created by weighting standardized inverse probability of treatment, in which we replaced extreme values of weight with those of the 1st and 99th percentiles. The Student t-test and Chi-square test were used to compare differences in means and proportions, respectively, of baseline covariates in the weighted sample. The covariates that were found to be different between the two groups with p-values less than 0.05 were further adjusted in the generalized linear model to compare treatment effects 30 days and 2 years after the operation. We calculated odds ratios (ORs) and the 95% confidence intervals (95% CIs) for the dichotomized indices**,** taking PF as the reference, and mean differences for the continuous variables adjusted for age, sex, and unbalanced variables. A two-sided p-value less than 0.05 was considered statistically significant. Statistical analysis was conducted using SAS version 9.4 (SAS Institute. Inc, Cary, NC, USA).

## Results

Table 2 shows demographic parameters, preoperative radiographical parameters**,** and QOL in the weighed sample (post propensity score matching) of patients undergoing LP or PF. There was no significant difference in age, sex, BMI, duration of symptoms, smoking history, or anticoagulant drug use. Concerning comorbidities, although there was no significant difference between the two groups with regard to diabetes mellitus, hypertension, malignancy, myocardial infarction, or collagen disease, the frequency of cerebrovascular disease was higher in the LP group than in the PF group (7.4% and 1.4%, respectively, p = 0.04). Baseline radiographical measurements were not significantly different between the groups, as assessed by confirming degree of cervical lordosis, ROM, K-line, thickness of ossification, spina canal occupation ratio of the OPLL > 60%, and increased signal intensity on MRI. Baseline functional status and QOL were also not significantly different, as assessed by JOA score or JOACMEQ. The two exceptions were the bladder function score in JOACMEQ, which was significantly higher in the PF group than in the LP group (mean (standard deviation, SD) of 79.7 (33.6) compared with 72.8 (20.5); p = 0.03) and VAS pain or numbness level in the arms or hands in JOACMEQ, which was significantly higher in the PF group than in the LP group (mean (SD) of 68.0 (32.8) compared with 59.9 (32.4); p = 0.048).

**Table 2:** Demographic parameters, preoperative radiographical parameters, and quality of life in the weighed sample of patients undergoing laminoplasty or posterior spinal fusion.

	Laminoplasty	Posterior spinal fusion	p
Age (year)	64.3 ± 11.9	66.7 ± 14.7	0.13
Sex (male ratio: %)	70.7	67.4	0.55
Body mass index	25.0 ± 3.7	24.8 ± 5.4	0.75
Smoking history (%)	33.2	37.2	0.50
Duration of symptoms (months)	44.4 ± 75.1	41.9 ± 81.7	0.80
**Levels of OPLL**			
C1–2 (%)	2.2	3.8	
C2–3 (%)	35.8	38.5	
C3–4 (%)	62.8	67.3	
C4–5 (%)	83.2	84.6	
C5–6 (%)	86.1	90.4	
C6–7 (%)	38.0	48.1	
C7–T1 (%)	6.6	13.5	0.86
Levels of surgery	3.5 ± 0.9	3.6 ± 2.3	0.96
**Comorbidities**			
Diabetes mellitus (%)	30.6	35.3	0.43
Hypertension (%)	41.6	36.9	0.45
Malignancy (%)	6.1	4.1	0.47
Cerebrovascular disease (%)	7.4	1.4	0.04
Myocardial infarction (%)	3.2	4.6	0.55
Collagen disease (%)	1.5	1.7	0.87
**Drug**			
Anticoagulant	11.8	7.2	0.21
**Radiographical measurements**			
Cervical lordosis (°)	11.6 ± 11.8	10.4 ± 14.4	0.42
Range of motion (°)	26.2 ± 12.7	25.6 ± 24.0	0.80
K-line (+/−) (%)	73.6	65.2	0.14
Thickness of ossification (mm)	5.1 ± 1.7	5.3 ± 2.8	0.34
Spinal canal occupation ratio > 60% (%)	8.6	13.2	0.24
Increased signal intensity on MRI (%)	86.2	92.0	0.14
JOA score	10.9 ± 2.6	10.6 ± 4.4	0.55
**JOACMEQ**			
Cervical function	61.8 ± 28.0	57.0 ± 44.1	0.24
Upper limb function	70.5 ± 22.3	65.1 ± 45.6	0.15
Lower limb function	54.9 ± 29.6	50.7 ± 47.4	0.35
Bladder function	72.8 ± 20.5	79.7 ± 33.6	0.03
Quality of life	44.0 ± 17.5	41.8 ± 32.6	0.43
**Visual analog scale**			
Pain or stiffness in the neck or shoulder	39.0 ± 33.0	41.4 ± 46.1	0.59
Tightness in the chest	9.7 ± 21.9	8.3 ± 26.2	0.63
Pain or numbness in the arms or hands	59.9 ± 32.4	68.0 ± 32.8	0.048
Pain or numbness from chest to toe	42.2 ± 34.4	47.5 ± 52.9	0.29

Summary statistics for continuous variables are means and standard deviations. *JOA* Japanese Orthopedic Association, *JOACMEQ* Japanese Orthopaedic Association Cervical Myelopathy Evaluation Questionnaire, *MRI* magnetic resonance imaging.

Overall, perioperative complications were significantly less common in the LP group than in the PF group (17.0% and 29.7%, respectively, p = 0.02). In particular, C5 palsy was significantly less frequent in the LP group than in the PF group (3.9% and 19.9%, p = 0.0002); however, there was no significant difference in dural tear, wound disruption, wound infection, or revision surgery. Multivariable logistic regression analysis showed that overall complications (OR 0.40, 95% CI 0.21–0.77, p = 0.006) and C5 palsy (OR 0.11, 95% CI 0.03–0.34, p = 0.0002) were significantly less common in the LP group than in the PF group (Table 3).

**Table 3:** Logistic regression analysis for complications and revision surgery in the weighted sample of patients undergoing laminoplasty or posterior spinal fusion.

	OR	95% CI	p
Overall complications	0.40	0.21–0.77	0.006
C5 palsy	0.11	0.03–0.34	0.0002
Dural tear	1.99	0.28–14.1	0.49
Wound disruption	0.26	0.02–3.62	0.32
Deep wound infection	0.54	0.11–2.60	0.44
Superficial wound infection	0.24	0.03–1.73	0.16
Revision surgery	0.31	0.01–11.10	0.52

Weighted for IPTW and further adjusted for baseline age, sex, VAS (pain or numbness in the arms or hands), preoperative JOACMEQ (bladder function), preoperative comorbidity (cerebrovascular disease).

*IPTW* inverse probability of treated weighting, *JOACMEQ* the Japanese Orthopaedic Association Cervical Myelopathy Evaluation Questionnaire, *OR* odds ratio, *CI* confidence intervals, *VAS* visual analogue scale.

Patients achieved similar postoperative functional and QOL outcomes**,** judging by JOA scores, JOA recovery rates, and JOACMEQ scores (Table 4). There were also no significant radiographical differences between the groups (Table 4). ROM and JOACMEQ cervical function score were significantly lower in the PF group than in the LP group (20.91 ± 1.05 and 9.38 ± 1.24, p < 0.0001; 67.64 ± 2.78 and 55.46 ± 3.29, p = 0.005, respectively) (Table 4). Multivariable logistic regression analysis showed no significant difference in the relationship between JOA score and MCID (JOA score > MCID in both groups) or JOA recovery rate and MCID (JOA recovery rate > MCID in both groups), or clinically significant improvement in JOACMEQ (Table 5). The percentage of patients with progression in the thickness of OPLL was significantly higher in the LP group than in the PF group (56.0% vs. 30.5% in PF; OR 2.73, 95% CI 1.60–4.67, p = 0.0002) (Table 5).

**Table 4:** Two years postoperative radiographical parameters and quality of life in the weighted sample of patients undergoing laminoplasty or posterior spinal fusion.

	Laminoplasty	Posterior spinal fusion	p
**Radiographical measurements**			
Cervical lordosis (°)	9.69 ± 1.10	7.66 ± 1.30	0.24
Range of motion (°)	20.91 ± 1.05	9.38 ± 1.24	< 0.0001
Thickness of ossification (mm)	6.11 ± 0.54	4.80 ± 0.64	0.12
JOA score	13.73 ± 0.21	13.72 ± 0.25	0.97
JOA RR	45.84 ± 3.08	51.15 ± 3.65	0.27
**JOACMEQ**			
Cervical function	67.64 ± 2.78	55.46 ± 3.29	0.005
Upper limb function	80.45 ± 1.76	77.53 ± 2.08	0.29
Lower limb function	63.44 ± 2.44	56.19 ± 2.89	0.058
Bladder function	77.19 ± 1.62	76.12 ± 1.92	0.67
Quality of life	53.63 ± 1.64	50.77 ± 1.94	0.27
**Visual analog scale**			
Pain or stiffness in the neck or shoulder	35.85 ± 2.78	38.18 ± 3.29	0.59
Tightness in the chest	9.94 ± 1.80	7.80 ± 2.13	0.89
Pain or numbness in the arms or hands	39.84 ± 2.70	44.20 ± 3.20	0.30
Pain or numbness from chest to toe	33.10 ± 2.83	34.78 ± 3.35	0.70

Values are means and standard**-**error. Values are weighted for IPTW and further adjusted for baseline age, sex, VAS (pain or numbness in the arms or hands), preoperative CMEQ (bladder function), preoperative comorbidity (cerebrovascular disease).

*JOA* Japanese Orthopaedic Association, *JOACMEQ* Japanese Orthopaedic Association Cervical Myelopathy Evaluation Questionnaire, *IPTW* inverse probability of treated weighting, *VAS* visual analogue scale.

**Table 5:** Logistic regression analysis for postoperative functional outcome and progression of OPLL in in the weighted sample of patients undergoing laminoplasty or posterior spinal fusion.

	OR	95% CI	p
JOA score > MCID	0.98	0.57–1.68	0.94
JOA RR > MCID	0.88	0.50–1.53	0.64
**Clinical improvement of JOACMEQ**			
Cervical function	1.05	0.58–1.87	0.88
Upper limb function	1.04	0.57–1.89	0.90
Lower limb function	1.75	0.89–3.44	0.11
Bladder function	1.16	0.59–2.28	0.67
Quality of life	1.88	0.95–3.70	0.07
Progression of OPLL	2.73	1.60–4.67	0.0002

Weighted for IPTW and further adjusted for baseline age, sex, VAS (pain or numbness in the arms or hands), preoperative JOACMEQ (bladder function), preoperative comorbidity (cerebrovascular disease).

*JOA* Japanese Orthopaedic Association, *JOACMEQ* Japanese Orthopaedic Association Cervical Myelopathy Evaluation Questionnaire, *OR* odds ratio, *CI* confidence intervals, *OPLL* ossification of the posterior longitudinal ligament, *MCID* minimum clinically important difference, *IPTW* inverse probability of treated weighting, *VAS* visual analogue scale.

## Discussion

This nationwide, multicenter, prospective study provides a comprehensive evaluation of the comparative efficacy of LP and PF for patients with cervical OPLL. There have been reports to date comparing laminoplasty and posterior fusion, but there have been only 3 prospective studies: Lee et al. (laminoplasty 21, posterior fusion 21), Liu et al. (laminoplasty 32, posterior fusion 35), and Bai et al. (laminoplasty 32, posterior fusion 32)^6^. Therefore, we thought it necessary to evaluate the results in a multicenter study. In addition, there have been no evaluations of QOL in this connection, and so we compared the JOACMEQ results as patient-reported outcome measurement (PROM) for cervical, upper limb, lower limb, bladder function, quality of life, and neck pain. The results indicated comparable improvement among patients with OPLL experiencing both surgical procedures at two years post-operation. The exceptions to this were the JOACMEQ cervical function scores and the cervical ROM on X-ray (Table 4), which were lower and smaller, respectively, in the patients with PF than in those with LP. In contrast, logistic regression analysis revealed no clinically significant difference in the improvement of JOACMEQ cervical function (Table 5), suggesting that the choice of PF or LP might have only a limited effect on the outcome for cervical function.

A relatively high incidence of surgical complications in cases of cervical OPLL compared with other forms of DCM has been reported; neurologic deficit and neck pain are particularly common in posterior procedures^4,26^. In the current study, the overall complication rate (OR 0.40) and the rate of C5 palsy occurrence (OR 0.11) were significantly lower with LP than with PF. There was no significant difference between the two techniques in the laterality of C5 palsy (16% of C5 palsy in laminoplasty cases was bilateral, but all other C5 palsy, including the C5 palsy that occurred with posterior fixation, was unilateral) or postoperative recovery (16% and 25% of patients experienced incomplete recovery in laminoplasty**-** and posterior fusion cases, respectively), and pathological differences in C5 palsy could not be clarified in this study. However, iatrogenic foraminal stenosis and larger posterior shift of the spinal cord might be associated with a higher risk of C5 palsy in PF^27,28^. Takemitsu et al. reported that the risk of postoperative C5 palsy with posterior instrumentation was 11.6 times greater than without instrumentation^29^, and this relative risk was similar to the current result. However, the actual incidence of C5 palsy after posterior procedures remains unclear**,** with some retrospective studies having reported it as 0%–30% and 2.6%–50% with LP and PF, respectively^27–31^. This prospective study, however, demonstrated that the risk of C5 palsy was 10 times higher with posterior instrumentation; thus, prophylactic foraminotomy might be recommended to reduce C5 palsy in cases with PF^30,31^. Since this study did not investigate whether or not prophylactic foraminotomy was performed, further investigation is needed to determine the usefulness of concomitant foraminotomy.

Neck pain was reported as another common postoperative complication in cases of cervical OPLL, and is more common in such cases than in cases of other forms of DCM^4^. Postoperative cervical kyphotic alignment might be associated with neck pain. Few studies have compared these complications between different posterior procedures in cases of cervical OPLL. However, in the present study, pain or stiffness in the neck or shoulder was comparable with both procedures (Table 4). Prior to the current study, we hypothesized that postoperative kyphotic alignment change was more frequent with LP than with PF, something which might be associated with postoperative neck pain. There were, however, no differences in this study in radiographical alignment or degree of postoperative neck pain between the two groups. Machino et al. reported that the kyphotic alignment change after laminoplasty was observed in 7.2% of 457 cases with preoperative lordotic alignment, and the incidence of postoperative kyphotic change was relatively low^32^. Although kyphotic change after LP was reported in another report^33^, cervical alignment is maintained in the majority of cases if patient selection is appropriate. It has also been reported that the angle of the T1 slope is important as an indicator of appropriate patient selection**,** i.e. for choosing patients who are less likely to display kyphotic changes after laminoplasty, but the relationship between T1 slope and postoperative kyphosis is still debated^33,34^. Recent studies have shown that postoperative kyphosis occurs in patients with low cervical extension function^35,36^. The risk of kyphosis may be higher in patients with extension range of motion (ROM) (extension—neutral C2-7 Cobb’s angle) of less than 14°^35^ or a gap ROM (flexion ROM—extension ROM) of greater than 27°^36^. Therefore, preoperative functional imaging of the cervical spine should be evaluated, and laminoplasty should be indicated in patients with a low risk of kyphosis, as reported by Lee and Fujishiro et al.^35,36^.

With respect to functional and QOL outcomes at two years post-surgery, there were significant differences in the average scores for the JOACMEQ cervical function domain section and cervical ROM on X-ray (Table 4). The JOACMEQ cervical function score is based on answers to four questions about daily life activities that require up-and-down movement and rotation of the neck^37^. PF was found to have reduced ROM, which might be associated with lower JOACMEQ cervical function score. Although JOACMEQ has criteria for determining whether these differences are clinically meaningful, multivariate analysis showed no significant differences in postoperative improvement of cervical function. Therefore, the effect on QOL was considered to be limited, although there was a numerical difference. Furthermore, although the perioperative complication of C5 palsy was more frequent in cases when PF was performed, the majority of C5 palsy cases showed improvement at two-years post-surgery. Therefore, it can be said that this complication was not associated with significant negative outcomes at two years post-surgery.

Although reoperation for the progression of OPLL after LP was not observed in the two postoperative years reviewed in the current study, revision surgery around 10 years after LP has been reported^15,16^. OPLL progression is commonly observed after LP^8,9^; 70% of patients in one study showed an increase in OPLL size 10 years after surgery^16^. The size of ossification commonly increases in young adults, those with continuous- or mixed-type OPLL, and those with kyphotic alignment change^15,16^. Takatsu et al. reported that mechanical stress after laminectomy may be the cause of OPLL progression^38^, and Ando et al. reported that micromotion at the site of ossification is involved in ossification progression, because fixation halted ossification progression^39^. Therefore, based on the current results as well, it is highly likely that micromotion after laminoplasty will promote ossification**,** and that fixation will inhibit ossification within the area of fixation. However, since there is a possibility that ossification may develop outside the area of fixation, further study is needed to determine the usefulness of the surgical technique in long-term follow-up.

## Strengths and limitations

The findings of this study are likely to be more generalizable than findings from single-center studies**,** since patients were prospectively enrolled at 24 multicenter sites. The large number of recruitment sites allowed us to evaluate outcomes for 189 patients with OPLL who received LP or PF. In addition, we evaluated outcomes using different radiographical and questionnaire tools, allowing for a comprehensive assessment of surgical outcomes in patients with OPLL.

This study has several limitations, however. First, a 26% attrition rate was observed at the two-year post-surgery. This was a result of the exclusion of cases with even one missing response to a quality-of-life questionnaire item, or missing x-ray or CT data. If clinical research coordinators could be appointed in each hospital to conduct detailed checks of the radiographical assessments and response results of research subjects, it would be possible to reduce the amount of missing data, but the high cost of doing so would be a major problem. Second, this is not an international study, and clinical outcomes might differ depending on race and ethnicity. Third, a standardized surgical protocol was not utilized across centers**,** and decisions about the approach, number of decompressed levels, and use of instrumentation and fusion were made at the discretion of the attending surgeon. Some baseline covariates were unbalanced between the two treatment groups in the weighted sample, and there may be residual systematic differences for other measured and unmeasured baseline covariates, which may yield a biased estimation of treatment effects. Although a backward elimination logistic regression model was used for baseline adjustment, PF was performed in more severe cases with less cervical lordosis or with larger spinal canal occupation of OPLL compared to LP. Therefore, a future prospective large-scale multicenter international study is needed to validate the current results. Fourth, the Open- and French-door laminoplasty techniques are grouped as the same laminoplasty in the current study, but complications and functional improvement may vary^20^. Although there was no significant difference in clinical outcomes and complications using either procedure in our previous paper which involved the same cohort^40^, future large cohort studies need a separate analysis for the two procedures. Fifth, the work in the present study, conducted at 28 academic institutions, was performed by highly experienced surgeons, but the number of years of experience of each of the surgeons is unknown. We cannot deny the possibility that the difference in experience of the surgeons between the two techniques may have affected the results. Lastly, in considering the usefulness of surgical treatment, a comparison with the natural course of cervical OPLL progression must be made. However, the present data set was not compared with data for the natural course. We are currently investigating the natural course of cervical OPLL in a multicenter study, which will make it possible to report on comparative results for quality of life in the future.

In conclusion, cervical LP and PF provide almost comparable functional and QOL improvements at two years after surgery, although perioperative complications were more numerous in cases where PF was performed.
